# Physiological and Molecular Responses of Barley Genotypes to Salinity Stress

**DOI:** 10.3390/genes13112040

**Published:** 2022-11-05

**Authors:** Omid Jadidi, Alireza Etminan, Reza Azizi-Nezhad, Asa Ebrahimi, Alireza Pour-Aboughadareh

**Affiliations:** 1 Department of Plant Breeding and Biotechnology, Science and Research Branch, Islamic Azad University, Tehran 14778-93855, Iran; 2 Department of Plant Breeding and Biotechnology, Kermanshah Branch, Islamic Azad University, Kermanshah 67187-73654, Iran; 3 Seed and Plant Improvement Institute, Agricultural Research, Education and Extension Organization (AREEO), Karaj 31585-854, Iran

**Keywords:** salt stress, barley, MGIDI discriminator, gene expression analysis, heatmap

## Abstract

Among cereals, barley is tolerant to high levels of salinity stress; however, its performance and global production are still dramatically affected by salinity. In this study, we evaluated the behavior of a set of advanced genotypes of barley with aim of assessing the physiological and molecular mechanisms involved in salinity tolerance. The experiment was conducted using a hydroponic system at optimal growing temperature and photoperiod conditions. The results of the analysis of variance (ANOVA) showed significant effects for salinity treatments and genotypes in terms of all measured traits. Salinity stress significantly increased the root and shoot Na+ contents and root-to-shoot Na^+^ and K^+^ translocations. In contrast, other physiological features, gas exchange-related traits, as well as root and shoot biomasses were significantly decreased due to salinity stress. Based on the results of the multi-trait genotype ideotype distance index (MGIDI) as a multiple-traits method, G12 and G14 were identified as the superior salt-tolerant advanced genotypes. In the molecular analysis, salinity stress significantly increased the mean relative expression of *HvSOS1*, *HvSOS3*, *HvHKT2*, *HvHKT3*, *HvNHX1*, and *HvNHX3* genes by 12.87-, 3.16-, 3.65-, 2.54-, 2.19-, and 3.18-fold more than the control conditions, respectively. The results of heatmap-based correlation and principal component analysis (PCA) revealed a clear association pattern among measured traits and expression data. Indeed, these associations confirmed relationships between tolerance pathways and physiological functions. In conclusion, the genotype G14 (D10*2/4/Productive/3/Roho//Alger/Ceres362-1-1) responded well to salinity stress and showed a better expression pattern of studied genes than other genotypes. Hence, this promising genotype can be a candidate for further assessments before commercial introduction.

## 1. Introduction

Barley (*Hordeum vulgare* L.), as one of the small-grain cereals, has a high tolerance to abiotic stresses such as salinity and drought. It is commonly used as a model plant to decipher salt-tolerance mechanisms due to its simpler genome than other cereal crops [1,2]. Based on FAO’s report, the global average of seed production and harvested area for barley are estimated ~3.50 tonh^−1^ and 504,000 hectares in 2020 [3]. As a whole grain, the kernels of this cereal have various health benefits such as providing minerals, fiber, protein, various types of vitamins, phosphorus, and calcium [4,5].

Owing to the appearance of climate change, abiotic stresses such as chilling injury, drought, salinity, and heat have devastating effects on agricultural production. Anecdotal evidence reveals that salt stress gently becomes a main limiting factor for plant growth and development, as well as an increase in a considerable loss of the productivity of crops. It is estimated that more than 800 million hectares of the world’s cultivated lands are affected by salinity [6,7]. However, despite escalating calls for more food in recent years, climate changes have caused increasing soil salinization in the wide areas around the world [8]. Hence, the development of salt-tolerant varieties can decrease the dramatic effects of salinity stress on crop production. In general, the accumulation of salt ions around the root and cells induces two types of stress, including osmotic and ionic. The osmotic stress appears when the accumulation of salt ions will be increased around roots which in turn water availability is limited for plant cells. On the contrary, ionic stress will be initiated through increasing the content of cytosolic chloride (Cl^−^) and Na^+^ in the developed leaves. Disruption of water transfer from the soil layers to the roots and followed by a decrease in the rate of shoot growth have been known as the two main consequences of these phenomena [9].

When plants encounter a high concentration of salts, several tolerance mechanisms are activated to maintain ion homeostasis in plant cells. The main of them is the regulation of the balance between potassium (K^+^) and Na^+^ accumulation in different tissues. Plants through this protective strategy exclude the extra Na^+^ and maintain the high concentrations of K^+^ for keeping a high intercellular K^+^:Na^+^ ratio and control ion homeostasis in plant cells [10]. Maintaining an optimal photosynthesis process is another key mechanism for inducing a high level of tolerance. Under salt stress conditions, the high accumulation of Na^+^ in cytoplasm results in a stomatal closure which in turn causes a remarkable imbalance between light capture through the photosystem II (PS II) and energy utilization. This event ultimately reduces the photosynthetic rate, disrupts the bio-energetic processes of photosynthesis, and induces the generation of reactive oxygen species (ROS) [11]. Furthermore, reduction in photosynthetic pigments under salt stress is a commonly revealed phenomenon, and in numerous studies, these features have been used as the important biochemical indicators of the cellular metabolic state [12]. There are several reasons for the decline in photosynthetic pigment contents and photosynthesis rate, so one of them is related to membrane deterioration [13]. In other words, the membrane stability index can be used as another key physiological indicator to screen salt-tolerant genotypes [2]. It has been reported that salinity stress significantly decreases the efficiency of PS II and the assimilation rate of CO_2_ [14]. Hence, the discovery of genetic materials with a high potential in terms of the efficiency of PS II can be worthwhile for utilization of them in breeding programs.

Progress in genetic and biological tools has resulted in the identification of numerous salt-responsive genes and transcription factors involved in inducing the salinity tolerance in plants. When plants undergo salt stress, the interaction among these genetic elements can form the basis for several pathways. As one of the important pathways, the SOS pathway (Salt Overly Sensitive) plays critical role in cellular ion homeostasis. The interactions between *SOS1*, *SOS2*, and *SOS3* genes form is the basis of this pathway. Indeed, higher *SOS1* activity in the root epidermis may be instrumental for Na^+^ exclusion from uptake, as well as mediates xylem Na^+^ loading under saline conditions [15]. The high-affinity potassium transporters (HKTs) are another important component involved in increasing salt tolerance in plants. During the stress period, the function of identified genes belonging to this group can remove Na^+^ from the xylem and finally increase the salinity tolerance [16]. Moreover, it has been known that Na^+^ influx is regulated by the sodium/hydrogen antiporter (NHX) family of cation/H^+^ transporters [17]. The *NHX* plays an important role in the transport of Na+ from the cytoplasm to the vacuole or outside of the cell; hence, the functions of *NHXs* genes can be effective in improving salt tolerance in plants [18].

Barley is an ideal model crop for studies on the mechanisms due to its high tolerance to this salinity stress. Hereby, evaluation of the physiological and molecular mechanisms could provide insight into trade-offs between various traits and transcriptomics aspects and also help to highlight key genes involved in salinity tolerance in barley. The main aims of the current study were to (i) identify of most tolerant barley genotypes based on a set of physiological traits at the seedling stage and (ii) investigate gene expression patterns for some genes involved in salinity tolerance in selected genotypes.

## 2. Materials and Methods

### 2.1. Plant Materials

In this study, a set of 19 advanced genotypes of barley along with a commercial cultivar (cv. Mehr), were investigated. All genotypes were provided from a national barley breeding program for the moderate climate in Iran. The pedigrees of the studied genotypes are shown in Table 1.

### 2.2. Physiological Assay at the Early Growth Stage

The experiment was conducted at the research glasshouse at the Cereal Research Department, Seed and Plant Improvement Institute (SPII), Karaj, Iran during 2019–2020. A hydroponic system with optimal growing conditions in terms of temperature (25 °C day, 20 ± 2 °C night) and photoperiod (16 h light, 8 h dark) was used to test genotypes at the seedling stage. All genotypes were planted on separate trays and transferred into buckets filled with 20 L of Hoagland nutrient solution [19]. The experimental materials were investigated through a randomized block design with three replications at two control (0 mM NaCl) and salinity stress conditions (200 mM NaCl). Aeration conditions to all buckets were supplied with a central air pump and several airstones and started 24 h after planting. A central air pump was used to supply optimal aeration conditions for all buckets. Furthermore, the nutrient solutions were changed every two days and their pH was controlled 2 times per day. At the third-leaf stage, salinity treatment was initiated by adding NaCl in five steps to reach 200 mM. After three weeks of applying the salinity treatment, seedling plants were subjected to sampling for physiological assays.

A handheld chlorophyll meter device (Minolta SPAD-502, Tokyo, Japan) was used to estimate the relative chlorophyll content (SPAD index). Several gas exchange parameters including net photosynthetic rate (PN), stomatal conductance (Gs), and transpiration rate (TE) were measured on the clean and healthy leaf using an infrared gas analyzer (LICOR, Lincoln, NE, USA). The membrane stability index (MSI) was measured according to a method proposed by Sairam et al. [20]. For this purpose, two sets of leaf samples were floated in 10 mL of double-distilled water. One set was maintained at 100 °C for 15 min in a boiling water bath and another at 40 °C for 30 min. The electronic conductivities (C1 for 40 °C and C2 for 100 °C) of each sample were recorded by a conductivity meter (AQUALYTIC, Dortmund, Germany). The membrane stability index was then calculated by the following mathematical relation:

MSI = [1 − (C1/C2)] × 100

The root and shoot Na^+^ and K^+^ concentrations were detected as proposed by Pour-Aboughadareh et al. [6]. Briefly, 10 mg of each sample was digested with 10 mL nitric acid (0.5 N) and maintained at 85 °C for 2 h in a boiling water bath. The digested samples were filtered and clear execrates were analyzed for Na^+^ and K^+^ using a flame photometry device (Sherwood Scientific Flame Photometer 420, Sherwood Scientific, Cambridge, UK). Moreover, the Na^+^ and K^+^ translocations from roots to shoots were estimated as proposed by Saqib et al. [21]:

Root-to-shoot Na^+^ translocation (RTSN) = [shoot Na^+^ content/root Na^+^ content]

Root-to-shoot K^+^ translocation (RTSK) = [shoot K^+^ content/root K^+^ content]

The roots and shoots fresh weights (RFW and SFW, respectively) were recorded through harvest the all seedlings of each genotype. Then, the samples were transferred into a hot air oven at 70 °C for 48 h to determine their dry weights (RDW and SDW, respectively).

#### Data Analysis

After obtaining physiological data, a combined analysis of the variance (ANOVA) was performed to test of the main effects (growth conditions and genotypes) and their interactions. The relative change (RC) due to salinity stress compared with the control conditions was calculated for all measured traits as used by Pour-Aboughadareh et al. [22]. The multi-trait genotype-ideotype distance index (MGIDI) was used to identifying the most tolerant genotype [23]. Accordingly, the genotypes with the lowest MGIDI scores are known as the ideal genotype. All statistical analyses were computed using R software [24].

### 2.3. Gene Expression Pattern Assay

To investigate six salinity-related genes (*HvHKT2*, *HvHKT3*, *HvNHX1*, *HvNHX3*, *HvSOS1*, and *HvSOS3*), the total RNA was isolated from selected tolerant barley genotypes (based upon physiological evaluation) with the help of DENAZIST ASIA kit (Tehran, Iran) according to the manufacturer’s instructions. A Nano-Drop Spectrophotometers device (Thermo Scientific-2000C, Waltham, MA, USA) was used to determine the concentration of the isolated RNA. Subsequently, cDNA was synthesized using EasyTM cDNA Synthesis Kit (Parstos, Tehran, Iran) per the manufacturer’s instructions. Each RT-qPCR reaction contained 6 μL of 2×RealQ Plus 2× Master Mix Green (Ampliqon, Odense, Denmark), 3.4 μL of RNAse-free water, 2 μL of cDNA (50 ng μL**^−1^**), and 0.3 μL of (0.3 L M) of each forward and reverse primers. The sequences and additional information for primers used in the expression study are shown in Table 2. All RT-qPCR reactions were run in a MiniOpticon^TM^ Real-Time PCR device (Bio-Rad, Hercules, CA, USA). Normalization of the expression of studied genes was carried out using the *a-tubulin* gene.

#### Statistical Analysis

The relative expression values of each gene were estimated using the CT values as suggested by Pfaffl [28]. Heatmap-based correlation and Principal component analysis (PCA) were computed to group the physiological traits and the relative gene expression data using R software [24].

## 3. Results

### 3.1. Effects of Salt Treatment on Physiological Traits

Based on the results of the analysis of variance (ANOVA), the main effect of salt treatment was significant for root and shoot fresh and dry weights (RFW, SFW, RDW, and SDW, respectively), relative chlorophyll content (SPAD), net photosynthetic rate (P_N_), stomatal conductance (Gs), transpiration rate (T_E_), and membrane stability index (MSI) traits. Significant differences were found for RFW, SFW, SDW, MSI, SPAD, and T_E_ among the evaluated advanced genotypes. The interaction effects between growth conditions and tested genotypes were significant only for SFW and T_E_ traits (Table 3). To detect the effect of salt stress on measured physiological traits, the relative change (RC) compared with the control condition for each trait was calculated. Accordingly, salinity stress significantly decreased G_S_, T_E_, SFW, SDW, RFW, and RDW by 82.96%, 81.19%, 76.63%, 60.44%, 61.03%, 60.44%, and 48.75%, respectively. Furthermore, a slight reduction was observed for MSI, and SPAD traits due to salt treatment (19.45%, 11.62%, and 8.35%, respectively) (Table 3).

### 3.2. Effects of Salt Treatment on Ionic Concentrations in Root and Leaf Tissues

According to the results of ANOVA, highly significant differences in salt treatments and genotypes, as well as their interactions were observed for the root and shoot Na^+^ and K^+^ concentrations (RN, SN, RK and SK, respectively), root and shoot K^+^:Na^+^ ratios (RKN and SKN, respectively), as well as root-to-shoot Na^+^ and K^+^ translocations (RTSN and RTSK, respectively) (Table 3). Salt treatment considerably increased the accumulation of Na+ content in both root and shoot tissues when compared with the control conditions (854.56% and 91.68%, respectively). On the contrary, the RK and SK characters significantly decreased by salt treatment (91.23% and 46.89%, respectively) across the 20 investigated barley genotypes (Table 3). In terms of RKN and SKN characters, salinity stress decreased these ratios by 96.11% and 94.70%, respectively, when they were compared with the control conditions (Table 3). Moreover, the mean of RTSN and RTSK significantly increased due to salt treatment in relative to the control conditions (356.27% and 677.46%, respectively) (Table 3).

### 3.3. Selection of Salt-Tolerant Genotypes Using All Measured Traits

**Figure 1:**
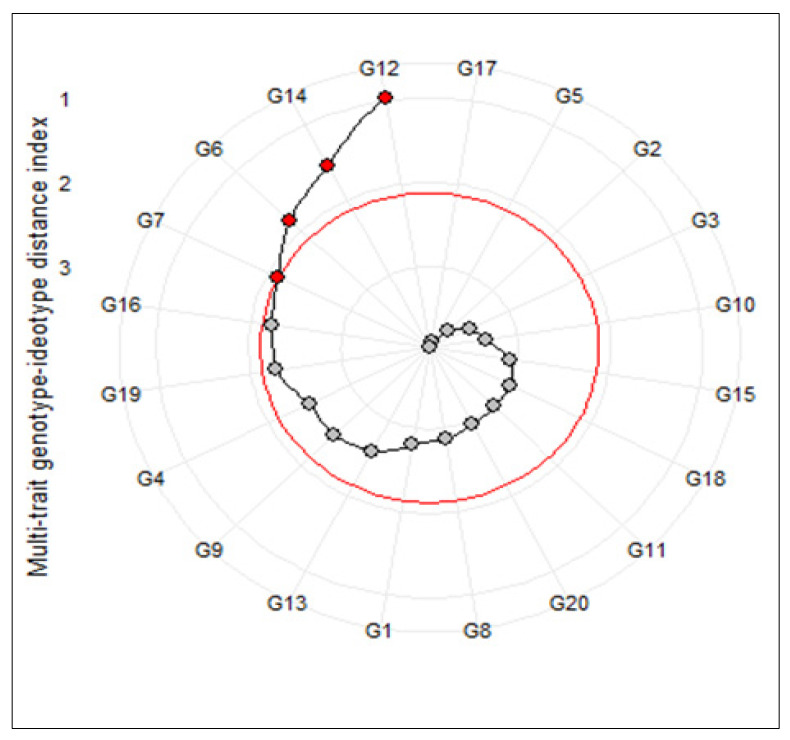
Genotype ranking in ascending order for the multi-trait genotype–ideotype distance (MGIDI) index. The selected genotypes based on this index are shown in red. The central red circle represents the cut-point according to the selection pressure.

The multi-trait genotype-ideotype distance index (MGIDI) was used to identify the superior salt-tolerant promising genotypes of barley. Based on the obtained results, nine traits including SFW, MSI, GS, RN, RK, SN, SK, RKN, and RTSN extracted using the first four main factors and indicated a highly significant genotypic effect in the MGIDI’s model (Table 4). The general heritability (h^2^) varied between 0.56 (RK) and 0.92 (SN). Indeed, all selected traits showed high heritability values, suggesting a strong capability of selection gains for these traits. Furthermore, the highest values for genetic gain were recorded by RN (19.60%), SN (9.06%), and RK (8.12%), respectively. On the contrary, MSI and RTSN showed an undesirable selection gain (−2.81% and −7.81%, respectively). Taking together, the main result of this method appeared as a circle plot that selected genotypes were highlighted by a red circle. As shown in Figure 1, the superior tolerant genotypes were G12, G14, G6, and G7. Hence, the first two genotypes were selected for further gene expression analysis.

### 3.4. Gene Expression Profiles

**Figure 2:**
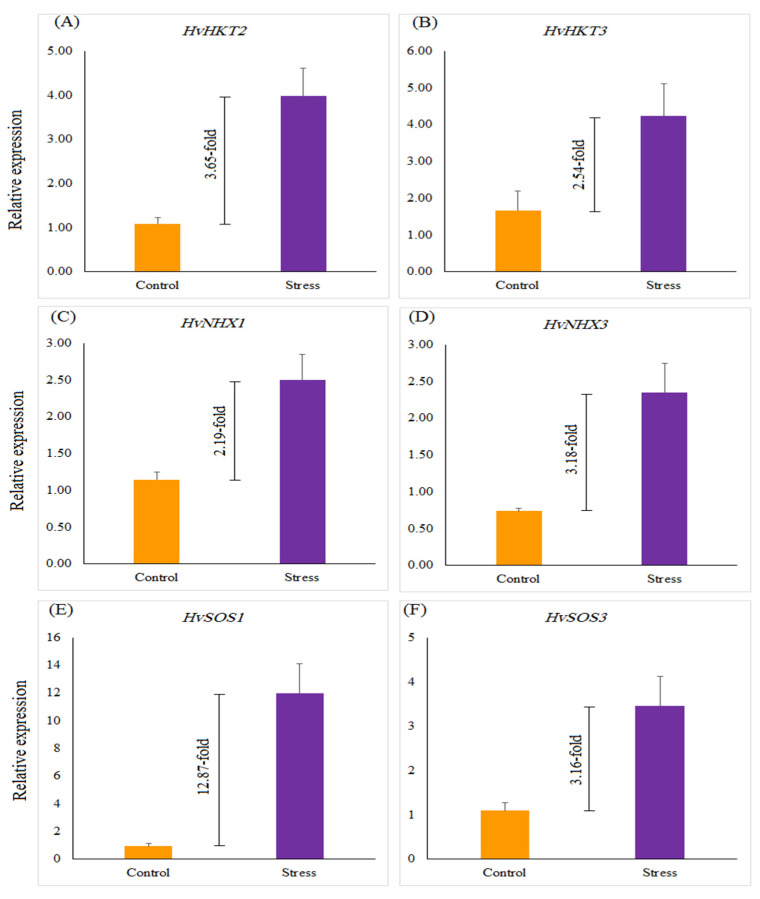
The relative expression of (**A**) *HvHKT2*, (**B**) *HvHKT3*, (**C**) *HvNHX1*, (**D**) *HvNHX3*, (**E**) *HvSOS1*, and (**F**) *HvSOS3* genes under the control and salinity stress conditions. Differences between control and stress treatments for all studied genes are significant at *p* < 0.01.

**Figure 3:**
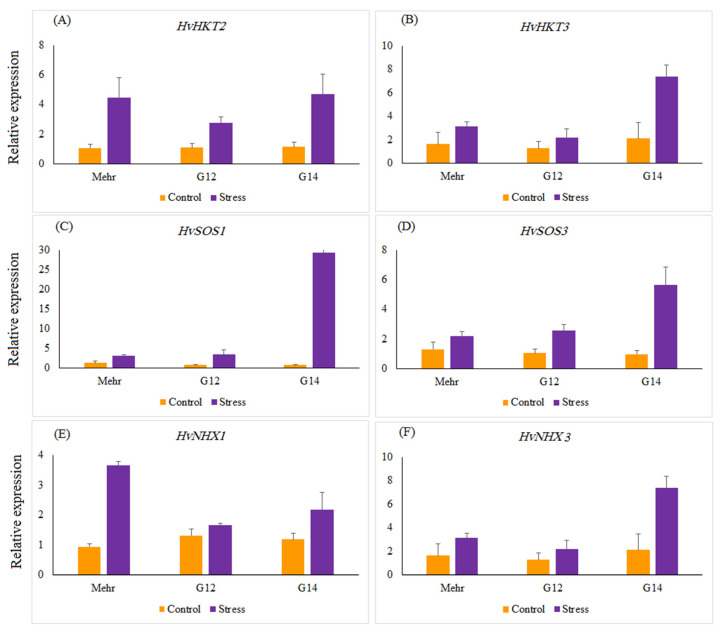
The relative expression patterns of (**A**) *HvHKT2*, (**B**) *HvHKT3*, (**C**) *HvSOS1*, (**D**) *HvSOS3*, (**E**) *HvNHX1*, (**F**) *HvNHX3* genes in selected barley genotypes under the control and salinity stress conditions. The two-way interaction between treatment and genotype main effects for all studied genes are significant at *p* < 0.01.

The relative gene expression of salinity-related genes, such as *HvHKT2*, *HvHKT3*, *HvNHX1*, *HvNHX3*, *HvSOS1*, and *HvSOS3*, revealed a significant difference in selected barley genotypes under salt stress treatment compared with the control. Salinity stress significantly up-regulated the expression of all genes by 12.87 (*HvSOS1*)-, 3.16 (*HvSOS3*)-, 3.65 (*HvHKT2*)-, 2.54 (*HvHKT3*)-, 2.19 (*HvNHX1*)-, and 3.18 (*HvNHX3*)-fold more than the control treatment (Figure 2). Under the control treatment, no significant difference was found among selected genotypes for the expression profiles of all genes, whereas in salinity treatment expressions of the studied genes were different from each other. Under salinity stress treatment, a striking increase in the relative expression of the *HvHKT2* gene was shown in the leaves of the Mehr cultivar (as a tolerant check cultivar; 4.22-fold) and the G14 (4.22-fold) and when compared with the control treatment (Figure 3A). Furthermore, the expression level of the *HvHKT3* gene in the G14 increased by salt treatment (3.55-fold compared to the control treatment), and this level of the transcript was more than the Mehr cultivar and another genotype (G12) (Figure 3B). Compared with the control treatment, the highest relative expressions of the *HvSOS1* and *HvSOS3* genes were found for the G14 genotypes (38.50-fold and 5.79-fold, respectively) than others (Figure 3C,D). In terms of the *HvNHX1* gene, the Mehr cultivar showed a higher number of transcripts (3.89-fold) than other selected genotypes (Figure 3E). However, the G14 revealed more relative expression of the *HvNHX3* (4.54-fold) compared with the Mehr cultivar (3.53-fold) and G12 (1.56-fold) under salinity stress conditions (Figure 3F).

### 3.5. Interrelationships between Physiological Traits and Gene Expression Data

**Figure 4:**
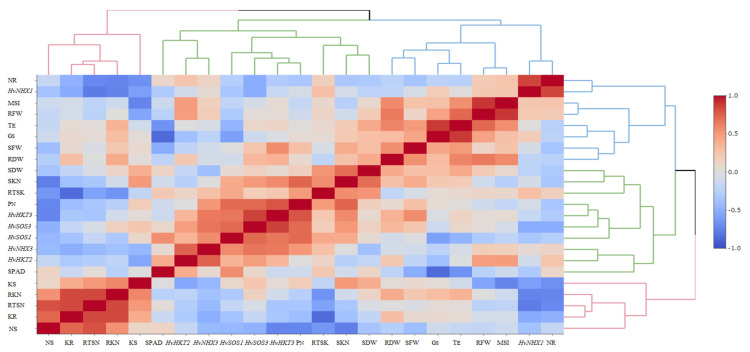
Correlation-based heatmap method reveals the association among measured traits and the relative gene expression patterns in the selected barley genotypes at the early growth stage. The different colors and intensities were adjusted based on associations among traits. RFW, root fresh weight; SFW, shoot fresh weight; SDW, shoot dry weight; SPAD, relative chlorophyll content; P_N_, photosynthetic rate; G_S_, stomatal conductance; T_E_, transpiration rate; MSI, membrane stability index; RN, root Na^+^ content; RK, root K^+^ content; SN, shoot Na^+^ content; SK, shoot K^+^ content; RKN, root K^+^:Na^+^ ratio; SKN, shoot K^+^:Na^+^ ratio; RTSN, root-to-shoot Na^+^ translocation; RTKN, root-to-shoot K^+^ translocation; *HvNHX1*, expression of the *HvNHX1* gene; *HvNHX3*, expression of the *HvNHX3* gene; *HvSOS1*, expression of the *HvSOS1* gene; *HvSOS3*, expression of the *HvSOS3* gene; *HvHKT2*, expression of the *HvHKT2* gene; *HvHKT3*, expression of the *HvHKT3* gene.

**Figure 5:**
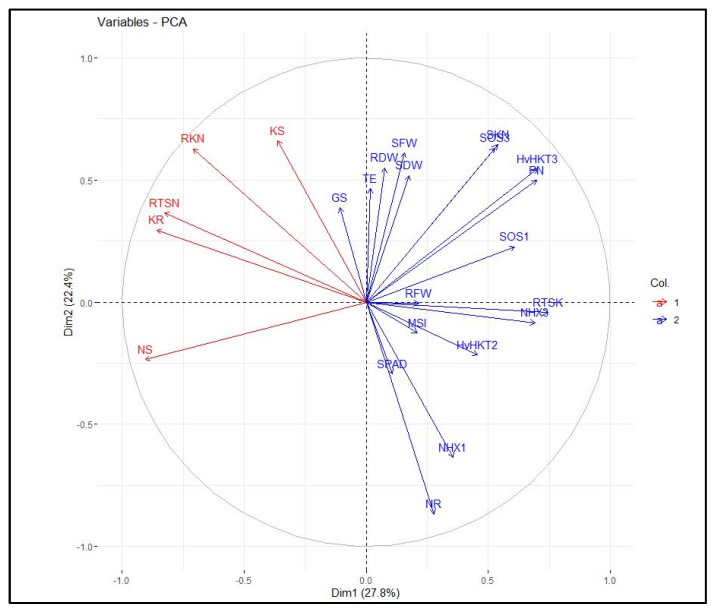
Principal component analysis (PCA) plot of measured traits and the relative gene expression data in the selected barley genotypes under salinity stress conditions. RFW, root fresh weight; SFW, shoot fresh weight; SDW, shoot dry weight; SPAD, relative chlorophyll content; P_N_, photosynthetic rate; G_S_, stomatal conductance; T_E_, transpiration rate; MSI, membrane stability index; RN, root Na^+^ content; RK, root K^+^ content; SN, shoot Na^+^ content; SK, shoot K^+^ content; RKN, root K^+^:Na^+^ ratio; SKN, shoot K^+^:Na^+^ ratio; RTSN, root-to-shoot Na^+^ translocation; RTKN, root-to-shoot K^+^ translocation; NHX1, expression of the *HvNHX1* gene; NHX3, expression of the *HvNHX3* gene; SOS1, expression of the *HvSOS1* gene; SOS3, expression of the *HvSOS3* gene; *HvHKT2*, expression of the *HvHKT2* gene; *HvHKT3*, expression of the *HvHKT3* gene.

The heatmap-based hierarchical cluster analysis (HCA) was performed with the aim of detecting the association among the relative expression of salt tolerance-related genes and measured traits. According to the obtained results, all physiological and molecular characters were grouped into two clear clusters. Accordingly, traits such as SN, RK, SK, RTSN, and RKN were grouped in the first cluster. The second cluster was further divided into two sub-clusters; the first included some traits such as SPAD, PN, SKN, SDW, and RTSK as well as the relative expression of *HvHKT2*, *HvHKT3*, *HvNHX3*, *HvSOS1*, and *HvSOS3* genes, and the second sub-cluster embraced RFW, RDW, SFW, GS, TE, MSI, RN, and relative expression of the *HvNHX1* gene. Based on the heatmap patterns represented in this dendrogram, there were positive and significant associations among each of the traits in the distinct clusters (Figure 4). Furthermore, the results obtained by the principal component analysis (PCA) indicated that the first two components (PC1 and PC2) accounted for 50.2% of the total variation. The PCA-based biplot also confirmed the grouping pattern of traits (Figure 5).

## 4. Discussion

In the present study, a set of plant growth-related and physiological traits was measured on some of the advanced genotypes of barley to evaluating their responses cope with severe salinity stress. As expected, there was observed a considerable effect of salt treatment and genotypic effects on the measured physiological traits (Table 3). Likewise, several studies confirmed the obtained results in the present study [2,29,30]. Under salinity stress conditions, aboveground tissues are the first organs that reveal damage from high salt concentrations in deeper soil layers. Indeed, when roots become drastically damaged due to salts, growth of the aboveground tissues are restricted through the disbalancing of nutrient and water uptake in roots. In this situation, osmotic stress is occurred, which turns the leaf area immediately reduced [2,31]. Our results revealed that root and shoot weights significantly decreased due to salinity stress when compared with the control conditions. As a result of absolute means comparison among genotypes, the genotypes G4 and G7 relatively indicated the lowest reduction of shoot fresh and dry weights, while in terms of root biomass there was a different reduction pattern among genotypes. Genotypes G11, G15, and G20 for root fresh weight, and G3, G14, and G20 for root dry weight showed the lowest reductions (Table S1). Previously, Pour-Aboughadareh et al. [2] and Ali and Abbas [32] reported a significant reduction in root and shoot weights of barley at the early stage of growth under different salt treatments.

Among plant physiological characteristics, the membrane stability index (MSI) is known as one of the most important indicators to assess potential salinity tolerance in a wide range of crops. Furthermore, Farooq and Azam [33] reported that this index is more effective in identifying tolerant genotypes at the seedling stage. It has been evidenced that this physiological character is affected by lipid peroxidation, which in turn results in the formation of malondialdehyde. (MDA) [34]. According to obtained results, salinity stress significantly reduced the MSI across all tested barley genotypes when compared with the control conditions (Table 3). These results revealed that there is a great genetic variation among tested genotypes in response to salinity stress, and G5, G10, and G18 showed the lowest reduction of MSI compared with other genotypes.

Chlorophyll and its related physicochemical processes, such as stomatal conductance, net photosynthesis process, and transpiration rate are the essential physiological events involved in plant health and development under adverse growth conditions. In the present study, the relative chlorophyll content and other photosynthesis-related traits showed reduction patterns due to salinity stress (Table 3). Although salinity stress significantly affected chlorophyll content, some genotypes showed a reverse pattern. To be more precise, the chlorophyll content slightly increased by salinity stress in genotypes G5 and G10. This result is further supported by Pour-Aboughadareh et al. [2], Higbie et al. [35], and Shin et al. [36]. In this regard, one of the main causes of increasing chlorophyll content can be related to as increasing salt solutions in the leaf, which in turn leads to a reduction of leaf area [37]. Moreover, Papp et al. [38] indicated a direct relation between increasing leaf thickness and reduction in leaf area; hence, this factor may be another case of increasing chlorophyll content under salinity conditions. In addition to the reduction in chlorophyll content, salt treatment significantly decreased the stomatal conductance (Gs) and transpiration rate (TE) parameters (Table 3). Regulation of gas exchange between the inner and outer space of the leaf is controlled by two symmetric guard cells. Stomatal conductance is one of the main phenomena involved in the regulation, which is controlled using several factors such as the density and status of its pore, as well as the ability of guard cells in the transport of water on the leaf surface [39]. The rate of CO_2_ is estimated using Gs, while the transpiration rate is determined by the degree of physical resistance to gas transport between the inner and outer space of the leaf [40]. In this research, the rate of reduction for Gs and TE was high; while salinity stress conditions did not significantly affect net photosynthesis (PN). These findings are in agreement with the reported results by Pour-Aboughadareh et al. [2], where a reduction in Gs and TE occurred due to salt treatment. Among investigated genotypes, G1, G12, and G18 showed the lowest reduction for Gs and TE, while the lowest reduction of PN was recorded in genotypes G4, G19, and G20 (Table S1).

Similar to biomass and physiological traits, ionic concentrations of root and leaf were changed due to salinity stress. One of the plant’s defense mechanisms to cope with the harmful effects of excessive Na^+^ is balancing between Na^+^ and K^+^ accumulation in different plant tissues [41,42]. Based on obtained results, salinity stress significantly declined the means of the content of K^+^ in the root and shoot tissues as well as the K^+^:Na^+^ ratios across all the evaluated barley genotypes (Table 3). The lowest reduction of root and shoot K^+^ contents was recorded in genotypes G8, G12, and G20, and genotypes G11, G17 and G20, respectively. In terms of root K^+^:Na^+^ ratio, genotypes G8, G11, and G20 showed the minimum reduction, while G7, G18, and G11 showed a minimum reduction for shoot K^+^:Na^+^ ratio than other genotypes (Table S1). Out of 20 investigated genotypes, two genotypes G7 and G18 indicated an increasing pattern for root K^+^ content, suggesting their excellent capability in excreting more Na+ and maintaining K^+^ content in their leaves. Although the mean root-to-shoot Na^+^ translocation (RTSN) increased by salinity stress (356.27% compared with control conditions), some genotypes including G20, G11, and G3, G17, and G12 showed a high ability to translocation of Na^+^. Salinity stress also increased the mean root-to-shoot K^+^ translocation (RTSK), and G18, G7, G17, and G11 were recognized as the superior barley genotypes in terms of transferring K^+^ ions to different organs.

During an experiment, researchers often prefer to gather various desirable plant growth characters and traits in a superior variety. On the other hand, identifying of superior genotype(s) based on various measured traits is often difficult. In some studies, researchers used various univariate and multivariate approaches such as ranking methods, cluster analysis, principal component analysis (PCA), various selection indices, and factor analysis to select superior genotypes [2,22,40,43,44,45,46,47]. Although these approaches can be useful in the selecting of the best genotypes, the direct and or indirect effects of some traits may be ignored. To solve this challenge, Olivoto and Nardino [23] recently developed a multi-trait genotype–ideotype distance index (MGIDI) as a novel multivariate approach for the selection of ideal genotype(s). In some studies, the use efficiency of this method was also confirmed [2,48,49,50]. According to this method, some measured traits including SFW, MSI, GS, RN, RK, SN, SK, RKN, and RTSN with a high heritability showed considerable ability for screening desirable barley genotypes at the seedling stage (Table 4). As a result obtained from this analysis, genotype G12 followed by G14, G6, and G7 were selected as the most salt-tolerant genotypes (Figure 1). For further dissection of tolerance of selected genotypes, we investigated expression patterns of several salt-tolerance-related genes in them. The result obtained from this section indicated that salt treatment increased the numbers of transcripts of *HvSOS1*, *HvSOS3*, *HvHKT2*, *HvHKT3*, *HvNHX1*, and *HvHKT3* genes than the control conditions. As a result, our findings revealed that G14 responded well to a high level of salinity stress compared to G12 and the check genotype.

Moreover, association analysis confirmed the close relationships among physiological and transcriptional aspects in the selected genotypes. For instance, there was a positive and significant association between the expression of *HvSOS1*, *HvSOS3*, *HvSOS1*, and *HvHKT3* genes with the shoot and root fresh and dry weights as well as gas exchange-related traits. The Salt-Overly-Sensitive (SOS) signaling transduction pathway is one of the main regulatory mechanisms for ion homeostasis and protecting different tissues and organs against high levels and persistence of salt [51]. In this pathway, three genes, including *SOS1*, *SOS2*, and *SOS3*, are involved, and their expressions are induced by salt stress [52]. It has been known that *SOS1*, *SOS2*, and *SOS3* have different expression profiles. The first two genes are expressed in both the root and shoot tissues, whereas the SOS3 is mainly expressed in root [53]. With increasing the salt around roots, Na^+^ enters the cortex, endodermis, and xylem tissues. Under this condition, Na^+^ compartmentalization and its loading to the xylem will be controlled by *SOS1*. In the present study, the results of PCA and heat-map-based cluster analysis confirmed that there is a positive correlation between root Na^+^ content with the expression of *HvSOS1*, *HvSOS2*, and *HvSOS3* genes (Figure 3 and Figure 4). Previously, Olias et al. [54] revealed a key role of *SOS1* in the loading of Na^+^ in the xylem. In this regard, our result also showed that the relative expression of *HvSOS1* negatively correlated with shoot Na+ content, while it showed a positive correlation with root Na^+^ content (Figure 4). Moreover, a weak correlation between the expression of *HvSOS3* and root Na^+^ content (Figure 4), confirming the role of this gene in controlling the exclusion of Na^+^ from root tissues.

In addition to the SOS pathway, the high-affinity K^+^ transporter (HKT) transporter is known as another critical mechanism for inducing salinity tolerance in plants. As is clear, the translocation and partitioning of Na+ inside plant tissues are two important factors that maintain plant health in saline conditions. After loading the Na^+^ at the root tissues, it will be transferred by some transporters to the xylem through several channels. In glycophyte plants controlling the translocation of Na^+^ from the root to shoot tissues is a key component in adaptation to saline environments [55]. Hence, *HKT* has been known as one of the main transporters that display specificity for Na^+^ and K^+^ [56]. These transporters can further be grouped into two sub-groups due to their transport selectivity. The first group is identified as Na^+^ uniporters, while the second group is identified as Na^+^–K^+^ symport [32]. Our results showed that there is a considerable association between the relative expression of *HvHKT2* and *HvHKT3* genes with root-to-shoot K^+^ translocation (RTSK) (Figure 4), indicating the importance of these genes in inducing salt tolerance in select tolerant barley genotypes. Furthermore, we observed a strong and positive association between the relative expressions of HKT and SOS genes. Indeed, this result is in accordance with the result reported by Ahmadi et al. [8], where a positive and significant correlation was observed between *HKT1* and *SOS1* genes.

A high cytoplasmic balancing between K^+^ and Na^+^ is regulated by various mechanisms such as morphological, physiological, and biochemical adaptations [9]. The Na^+^/H^+^ antiporter (*NHX*) is another intercellular transporter that widely exists in all organisms. These genes are key in stoma regulation, osmotic regulation, and flower development [57,58]. The *NHX* family can be classified into two distinct classes: the first class includes NHX isoforms of Arabidopsis thaliana (*AtNHX1-4*) with strong vacuolar localization, and the second class includes NHX proteins (*AtNHX5-6*) that display endosomal localization in cells [59,60,61]. For barley, four isoforms for the *NHX* family including *HvNHX1*, *HvNHX2*, *HvNHX3*, and *HvNHX4* are identified and mainly localized in the vacuole [62]. In general, the expression of these genes under salt stress depends on the degree of salt sensitivity and even species [62]. In this research, salt treatment remarkably increased the relative expression of *HvNHX1* and *HvNHX3* genes when compared with the control conditions (Figure 1). Moreover, the numbers of transcripts of the *HvNHX3* gene positively correlated with *SOS* genes and some physiological traits such as shoot K^+^:Na^+^ ratio, root Na^+^ content, root-to-shoot K^+^ translocation, as well as root and shoot biomasses (Figure 4). These results indicated that a strong trade-off between the expression of key pathways involved in salinity tolerance leads to inducing a high level of salinity tolerance in the barley seedlings, especially genotype G14.

## 5. Conclusions

The development of new superior varieties with an impressive tolerance to salinity stress needs a comprehensive investigation of various growth aspects and molecular mechanisms. In this regard, the early growth stage provides a better opportunity to investigate the physiological characteristics of genotypes. Our results revealed a high level of genetic variation in response to salinity stress for various physiological traits among a set of breeding barley genotypes at the early growth stages. The MGIDI index as a novel multiple-based traits discriminator identified several most tolerant genotypes for further molecular investigation. The results of RT-qPCR showed that salinity stress increased the relative expression of *HvSOS1*, *HvSOS3*, *HvHKT2*, *HvHKT3*, *HvNHX1*, and *HvNHX3* genes compared with the control conditions. Taken together, our results revealed that the genotype G14 can be a candidate as a superior salt-tolerant barley genotype for further experiment before commercial introduction.

## Figures and Tables

**Table 1:** The pedigrees of the 20 investigated barley genotypes.

Code	Pedigree
G1	Mehr cultivar (Check)
G2	Karoon /4/(D-13)Bgs/Dajia//L.1242/3/(L.B.IRAN/Una8271//Gloria’S’/3/Alm/Una80//....)
G3	(D10)Rhn-03//L.527/NK1272/ /(D-16)Bda/Rhn-03//ICB-107766
G4	(Salt-4) LB.Iran/Una 8271//Gloria”S”/Come”s”-11M/3/Kavir/4/Karoon
G5	(Salt-4)LB.Iran/Una 8271//Gloria”S”/Come”s”-11M/3/Kavir/4/(Salt12)ROHO/MAZORKA//TROMPILO
G6	(Salt-12)ROHO/MAZORKA//TROMPILO/3/Lignee 527/NK1272//JLB 70-63
G7	Lignee 527/NK1272//JLB 70-63/3/Zarjow
G8	KAROON/KAVIR//Rhodes’S’//Tb/Chzo/3/Gloria’S’/4/Zarjow
G9	KAROON/KAVIR//Rhodes’S’//Tb/Chzo/3/Gloria’S’/4/Karoon
G10	Bgs/Dajia//L.1242/3/(L.B.IRAN/Una8271//Gloria’S’/3/Alm/Una80//)/4/Nimrooz
G11	(D-16)Bda/Rhn-03//ICB-107766/3/Yousef
G12	Deir Alla 106//Hem/Bc/3/Rihane”s”14/4/Lignee527/NK1272//JLB70-063/3/Barjouj
G13	Yousef*2/3/Np106/Minn14133-Gvaxduois//Gi10143
G14	D10*2/4/Productive/3/Roho//Alger/Ceres362-1-1
G15	D10*2/4/Productive/3/Roho//Alger/Ceres362-1-1
G16	Lignee 527/NK1272//JLB 70-63/5/CLN-B/80.5138//GLORIA-BAR/COPAL/3/ALISO/4/CABUYA/6/(D10)Rhn-03//L.527/NK1272
G17	Courlis/Rhn-03//Productive/3/Lignee 527/NK1272//JLB 70-63
G18	Comp.Cr229//As46/Pro/3/Srs/4/Express/5/Rhn-03 * 2/M83-194 Ras3 * 2/6/Lignee 527/NK1272//JLB 70-63
G19	26216/4/Arar/3/Mari/Aths * 2//M-Att-73-337-1/5/Fajr30/6/Lignee 527/NK1272//JLB 70-63
G20	POA/Hjo//Quina/3/Rojo

**Table 2:** The sequences of the used selected salt-tolerance-related genes.

Gene	Forward/Reverse	Sequence	Reference
*HvHKT-2*	F	GACCCTTTCTCCACCGATTA	[25]
	R	CACGAGCCGATTTACACG	
*HvNHX1*	F	TGCATATCTACCAGTGCTTAT	[26]
	R	GGTTCAAGACACAAGTTCAGT	
*HvNHX2*	F	GGTTTTCGGCTTGCTGACTAA	[26]
	R	CATTGGGCGCATGAACTTATC	
*HvNHX3*	F	TGAGCCGAACATTACTGTGAT	[26]
	R	ACGAGCTTACCTTTCAATACA	
*HvSOS1*	F	GGCACCAACAGGAAGATGAA	[27]
	R	GATATGCAGGAGGCCAGAGA	
*HvSOS3*	F	GCTGCACCTCGAAAATCC	[27]
	R	AAACCGCTCGTCACTGCT	
*a-tubulin*	F	AGTGTCCTGTCCACCCACTC	[25]
	R	ATTCAGAGCACCGTCAAACC	

**Table 3:** The level of probability and mean values for each measured trait in 20 barley genotypes under control and salinity stress conditions.

Trait	Salinity Treatment (S; *df = 1*)	Genotype (G; *df = 19*)	S × G (*df = 19*)	Mean	
				Control	Stress
Root fresh weight (RFW)	***	**	ns	3.15	1.24
Shoot fresh weight (SFW)	***	**	*	18.30	4.28
Root dry weight (RDW)	**	ns	ns	0.26	0.13
Shoot dry weight (SDW)	**	*	ns	2.41	0.94
Membrane stability index (MSI)	*	***	ns	90.24	72.69
Relative chlorophyll content (SPAD)	*	**	ns	38.20	33.77
Photosynthetic rate (P_N_)	ns	ns	ns	23	21.08
Stomatal conductance (*G_S_*)	*	ns	ns	19.35	3.30
Transpiration rate (*T_E_*)	***	**	**	0.82	0.15
Root Na^+^ content (RN)	***	***	***	126.45	242.38
Shoot Na^+^ content (SN)	***	***	***	12.30	117.38
Root K^+^ content (RK)	***	***	***	9.71	0.85
Shoot Na^+^ content (SN)	***	***	***	12.79	6.79
Root K+/Na+ ratio (RKN)	***	***	***	0.10	0.004
Shoot K+/Na+ ratio (SKN)	***	***	***	1.15	0.06
Root-to-shoot Na^+^ translocation (RTSN)	***	***	***	0.12	0.53
Root-to-shoot K^+^ translocation (RTSK)	**	*	*	1.43	11.09

ns, Non-significant; *, *p* < 0.05; **, *p* < 0.01; ***, *p* < 0.001.

**Table 4:** Predicted genetic gain for the effective traits in the MGIDI index under salinity stress conditions.

Factor	Trait	Goal	*h^2^*	Selection Gain (%)
FA1	Root K^+^ content	Increase	0.56	8.12
FA1	Root K^+^:Na^+^ ratio	Increase	0.65	3.08
FA1	Shoot Na^+^ content	Decrease	0.93	9.06
FA1	Root-to-shoot Na^+^ translocation	Decrease	0.87	−7.81
FA2	Membrane stability index	Increase	0.63	−2.81
FA2	Shoot fresh weight	Increase	0.77	0.64
FA2	Shoot K^+^ content	Increase	0.61	0.03
FA3	Stomatal conductance	Increase	0.61	0.15
FA4	Root Na^+^ content	Decrease	0.73	19.60
Total (Increase)				12.02
Total (Decrease)				7.46

## Data Availability

Not applicable.

## Supplementary Materials

The following supporting information can be downloaded at: https://www.mdpi.com/article/10.3390/genes13112040/s1, Table S1: The mean values of measured traits in barley genotypes at the seedling stage under control and salinity conditions.
