# Supplementary material for: Physiological and Molecular Responses of Barley Genotypes to Salinity Stress
Source: Genes (Basel). 2022 Nov 5;13(11):2040. doi: 10.3390/genes13112040 (PMC9690512; doi:10.3390/genes13112040)
Supplement: Supplementary file 1 [file genes-13-02040-s001.zip › genes-2001518-supplementary.pdf]

Table S1. The mean values of measured traits in barley genotypes at the seedling stage under control and salinity conditions.

Genotype code	RFW		RDW		SFW		SDW		MSI		SPAD		P_N		G_S		T_E	
	C	S	C	S	C	S	C	S	C	S	C	S	C	S	C	S	C	S
G1	3.08	1.65	0.27	0.12	19.03	3.55	2.88	0.83	92.03	78.72	38.73	33.60	20.90	16.87	5.33	5.00	0.32	0.30
G2	4.58	1.37	0.31	0.20	24.17	7.63	2.97	1.26	73.76	37.66	39.33	33.67	22.00	20.63	15.40	7.00	0.31	0.09
G3	3.48	0.90	0.15	0.10	24.39	6.63	2.05	1.04	87.90	80.46	36.20	26.93	26.87	21.43	18.35	0.63	1.08	0.05
G4	4.13	1.35	0.30	0.13	11.90	4.55	2.30	1.34	92.53	79.36	37.40	36.90	22.10	17.70	18.27	3.60	0.36	0.16
G5	3.43	1.10	0.30	0.13	17.20	4.17	2.56	0.87	93.36	92.89	37.00	38.30	21.80	18.03	34.33	0.97	0.48	0.07
G6	3.80	1.08	0.30	0.12	29.07	6.17	3.69	0.96	89.03	66.03	37.13	35.60	23.97	17.90	49.00	2.03	1.24	0.17
G7	2.60	1.27	0.22	0.14	14.20	7.05	2.01	1.07	89.85	77.47	38.63	30.30	28.73	24.57	44.33	4.30	1.45	0.23
G8	2.92	1.42	0.28	0.12	18.03	3.12	1.75	0.83	93.05	76.29	38.60	33.50	25.10	18.73	30.48	1.86	0.68	0.11
G9	2.90	1.53	0.31	0.16	23.72	4.95	2.76	1.01	92.60	74.89	35.37	27.67	20.90	15.05	14.60	1.95	1.59	0.08
G10	4.70	1.75	0.40	0.18	21.75	5.47	2.52	1.00	91.72	86.66	37.80	39.60	29.00	19.80	20.67	1.85	1.72	0.03
G11	1.92	1.08	0.18	0.12	19.43	4.33	2.39	0.99	91.05	77.98	42.40	30.17	25.17	20.47	19.70	2.50	0.34	0.15
G12	2.38	1.18	0.19	0.13	16.03	3.77	2.37	0.84	93.16	57.51	36.10	34.33	21.43	20.13	7.93	4.76	0.44	0.26
G13	3.27	0.95	0.30	0.11	16.42	3.60	2.44	0.82	88.83	57.96	37.90	33.80	24.00	22.53	10.47	1.60	0.65	0.09
G14	2.33	1.22	0.20	0.14	13.75	4.25	2.06	0.76	89.70	61.03	42.07	34.97	24.40	23.67	15.97	3.55	0.88	0.18
G15	1.60	0.87	0.22	0.10	9.63	1.98	1.84	0.67	91.08	75.98	37.97	36.93	29.15	20.50	20.47	3.00	1.13	0.17
G16	3.30	1.30	0.29	0.13	19.47	3.60	2.47	1.17	91.56	81.89	38.07	34.63	26.80	19.37	27.27	5.75	1.53	0.22
G17	2.60	0.63	0.24	0.07	16.67	2.40	2.31	0.55	89.76	72.56	38.50	33.90	21.93	19.83	13.07	2.00	0.89	0.14
G18	2.18	1.10	0.20	0.10	14.20	2.60	2.10	0.68	93.81	90.66	42.97	36.03	25.20	23.57	8.50	6.49	0.37	0.36
G19	4.38	0.93	0.25	0.11	17.50	2.33	1.92	0.66	92.60	51.98	38.30	33.40	17.33	16.77	6.73	2.33	0.34	0.15
G20	3.33	2.20	0.24	0.23	19.42	3.38	2.72	1.41	91.91	75.33	33.60	31.07	24.25	23.10	8.15	2.77	0.64	0.10
Means	3.15	1.24	0.26	0.13	18.30	4.28	2.41	0.94	90.24	72.69	38.20	33.77	23	21.08	19.35	3.30	0.82	0.15

RFW, root fresh weight (g $plant^{-1}$); RDW, root dry weight (g $plant^{-1}$); SFW, shoot fresh weight (g $plant^{-1}$); SDW, shoot dry weight (g $plant^{-1}$); MSI, membrane stability index; SPAD, relative chlorophyll content (SPAD value); P_N, photosynthesis rate(μmol CO_2 m^{-2} $s^{-1)}$, $G_{S,}$ stomatal conductance (μmol H_2O m^{-2} s^{-1}), $T_{E,}$ transpiration rate (μmol H_2O m^{-2} s^{-1})

C, control conditions; S salinity stress conditions; R%, percent reduction due to salinity stress compared to control conditions (negative numbers indicate value higher than control conditions)

Table S1. Continued.

Genotype code	RN		SN		*RK*		*SK*		RKN		SKN		RTSN		RTSK	
	C	S	C	S	C	S	C	S	C	S	C	S	C	S	C	S
G1	72.45	347.54	9.99	120.89	10.67	0.60	14.87	6.24	0.15	0.002	1.48	0.06	0.15	0.35	1.48	12.59
G2	105.01	184.10	13.70	65.18	9.19	0.66	13.92	3.70	0.09	0.004	1.12	0.06	0.14	0.37	1.54	10.24
G3	118.68	204.14	10.35	127.88	14.90	1.15	12.73	5.35	0.13	0.016	1.29	0.04	0.09	0.69	0.86	4.63
G4	79.45	278.57	10.85	123.03	14.01	1.49	13.42	5.28	0.19	0.015	1.26	0.04	0.14	0.44	0.96	7.63
G5	121.73	177.50	10.84	108.80	8.73	0.64	14.23	7.88	0.09	0.004	1.57	0.07	0.13	0.66	1.64	17.36
G6	86.84	300.42	11.34	121.47	13.48	0.87	16.16	5.89	0.17	0.003	1.48	0.05	0.14	0.41	1.24	13.20
G7	116.06	309.30	12.63	129.57	11.08	0.71	3.42	5.33	0.11	0.002	0.30	0.05	0.14	0.44	0.33	7.27
G8	285.88	270.38	14.23	72.30	5.60	0.69	15.86	7.08	0.02	0.003	1.13	0.10	0.05	0.27	2.84	10.20
G9	74.67	241.78	18.03	105.70	12.16	0.84	16.77	7.51	0.17	0.004	1.05	0.07	0.25	0.47	1.41	9.36
G10	74.88	293.59	8.88	98.11	9.70	1.02	13.42	6.50	0.14	0.004	1.48	0.07	0.13	0.33	1.48	6.98
G11	275.24	155.65	12.63	123.20	6.64	0.59	7.54	6.78	0.02	0.004	0.62	0.06	0.05	0.82	1.13	12.41
G12	133.26	245.11	14.55	155.94	8.93	1.78	16.47	8.56	0.07	0.01	1.18	0.05	0.11	0.64	1.92	5.11
G13	152.85	238.28	14.34	125.70	7.99	0.75	12.24	7.70	0.05	0.003	0.86	0.06	0.10	0.54	1.52	14.06
G14	103.15	261.49	9.78	112.27	9.11	0.91	12.50	7.67	0.09	0.004	1.37	0.07	0.10	0.43	1.37	10.55
G15	90.54	215.52	7.99	59.84	7.36	0.62	11.43	5.01	0.08	0.003	1.50	0.08	0.09	0.28	1.72	8.41
G16	131.37	302.93	16.90	124.08	7.20	0.71	13.87	5.77	0.06	0.002	0.83	0.04	0.13	0.42	1.96	9.75
G17	110.57	179.55	10.49	122.96	8.00	0.35	10.46	9.35	0.08	0.002	0.99	0.08	0.12	0.77	1.31	28.69
G18	100.62	234.18	11.70	102.05	10.08	0.49	5.93	8.37	0.10	0.002	0.57	0.08	0.12	0.46	0.63	16.96
G19	147.03	241.01	18.72	128.59	9.26	0.84	15.32	6.12	0.07	0.003	0.82	0.05	0.14	0.58	1.66	8.71
G20	148.76	166.58	7.99	220.03	10.14	1.31	15.25	9.76	0.07	0.01	2.00	0.04	0.06	1.33	1.54	7.72
Mean	126.45	242.38	12.30	117.38	9.71	0.85	12.79	6.79	0.10	0.004	1.15	0.06	0.12	0.53	1.43	11.09

RN, root Na^+ content; SN, shoot Na^+ content; RK, root K^+ content (mmol g^{-1} DW); SN, shoot Na^+ content (mmol g^{-1} DW); SK, shoot K^+ content (mmol g^{-1} DW); RKN, root K^+:Na^+ ratio; SKN, shoot K^+:Na^+ ratio; RTSN, root-to-shoot Na^+ translocation; RTKN, root-to-shoot K^+ translocation

C, control conditions; S salinity stress conditions; R%, percent reduction due to salinity stress compared to control conditions (negative numbers indicate value higher than control conditions)
